# Methionine to cystine ratio in the total sulfur amino acid requirements and sulfur amino acid metabolism using labelled amino acid approach for broilers

**DOI:** 10.1186/s12917-018-1677-8

**Published:** 2018-11-22

**Authors:** Letícia G. Pacheco, Nilva K. Sakomura, Rafael M. Suzuki, Juliano C. P. Dorigam, Gabriel S. Viana, Jaap Van Milgen, Juliana C. Denadai

**Affiliations:** 1 Department of Animal Science, Unesp, Jaboticabal, SP Brazil; 2 Evonik Nutrition & Care GmbH, Hanau, Germany; 3 INRA, Agrocampus Ouest, Rennes, France; 4 Department of Animal Science, Unesp, Botucatu, SP Brazil

**Keywords:** Cystine, Methionine conversion, Methionine cycle, Stable isotopes, Sulfur amino acids

## Abstract

**Background:**

Assuming that part of Methionine (Met) is converted into Cystine (Cys), but ignoring the rates with which such phenomenon occurs may lead to an excessive supply of Met in poultry diets. Such inconvenient could be easily avoided with the knowledge of the ideal Met:Cys/Total sulfur amino acids (TSAA) ratio and the rates of Met conversion into Cys.

**Results:**

Met sources did not affect performance. Met:Cys/TSAA ideal ratio was determined using curvilinear-plateau regression model. Both optimum body weight gain and feed conversion ratio were estimated in 1007 g/day and 1.49, respectively, at 52% Met/TSAA ratio. Feed intake was not affected by Met:Cys/TSAA ratios. In the labelled amino acid assay, the rates with which Met was converted into Cys ranged from 27 to 43% in response to changes in Met:Cys/TSAA ratios, being higher at 56:44.

**Conclusion:**

Based on performance outcomes, the minimum concentration of Met relative to Cys in diets for broilers from 14 to 28 d of age based on a TSAA basis, is 52% (52:48 Met:Cys/TSAA). The outcomes from labelled amino acid assay indicate that highest the Met supply in diets, the highest is its conversion into Cys.

## Background

The composition of feedstuffs used in feed production and the amount required for different metabolic functions makes methionine (Met) more limiting in poultry diets as compared to other essential amino acids [1]. Except for tryptophan in corn protein, Met is the amino acid, whose concentration is smallest one among essential amino acids in the protein of corn and soybean meal [2]. Contrary to lysine (Lys), which is used almost entirely for body protein synthesis and does not take part in other metabolic processes [3], or threonine (Thr) and valine (Val), whose intake is deviated for few secondary functions like intestinal mucin protein synthesis [4], Met is less efficiently retained into body due to its role as a donor of methyl groups in the synthesis of compounds such as creatine, choline, polyamines and carnitine [3].

Poultry are not capable of synthetizing the carbon skeleton of Met in a sufficient amount to meet the relative needs of the organism for maintenance and growth; and therefore are completely dependent on its dietary supply to ensure the ideal rate for protein synthesis [5, 6]. An extremely important function of Met in animal metabolism, is as the precursor of cysteine [7]. As well as Met, cysteine is a sulfur amino acid formed via a transsulfuration pathway in the hepatocytes, whose synthesis requires a thiol group from Met and a nitrogen atom from serine. Due to its instability, cysteine forms disulphide bonds with another cysteine molecule to originate cystine (Cys), which confers strength and stiffness to keratin [8, 9]. Proportionally, feather proteins contain more Cys than most other tissue proteins [10, 11]. Emmans and Fisher [12] describe Met content in the protein of broiler carcass as being approximately two-fold higher than Cys content (2.5 vs. 1.1%), whilst in feathers such proportion markedly changes and Cys assumes a higher participation in protein composition (approximately twelve-fold higher) compared with Met (7 vs. 0.6%). Similarly, Talpaz et al. [13] reported that Cys content in the protein of broiler feathers was thirteen-fold higher than Met (6.5 vs. 0.5), but contrary to the aforementioned authors, found a different proportion between TSAA in carcass, whose value of Cys content was higher than Met (2.4 vs. 1.9%).

Based on such background about the role of Cys on feather composition and on the fact that broiler feathering rates exhibit different patterns of growth throughout growing phase, reaching the maximum rate at approximately 28 d post-hatch, it is expected that bird requirement for Cys follows the same behavior of feather growth. Commercial diets are traditionally formulated to meet broiler requirements for methionine + cystine (Met + Cys), based on the assumption that amounts of dietary Met are converted into Cys. The consequence is that Met may be provided in excess, reducing the efficiency with which the amino acid is used by the organism. This inconvenient could be easily avoided with the knowledge of the ideal Met:Cys/TSAA ratio and the amounts of Met, which are converted into Cys. Since Cys synthesis is dependent on Met, providing broiler diets supplemented with L-cystine could may spare the utilization of Met, which could increase its availability on organism for body protein accretion, and hence improve broiler performance and mitigate nitrogen excretion. Several research efforts has been made to establish broiler requirements for Met, which in turn, allowed the TSAApublication of several feed tables. Brazilian Tables for Poultry and Swine [2], recommend TSAAthe optimum daily intake of digestible Met for male broilers in 130, 401, 786, and 938 mg/bird, in the pre-starter, starter, grower, and finisher phases, respectively, whilst daily digestible Cys requirements for the these phases is described as 105, 323, 633, and 755 mg/bird.

Little is known about the ideal Met:Cys/TSAA ratio for broilers, feed tables recommend Met:Cys/TSAA ratio of 55:45 [2, 14]. With regard to Met sources, poultry diets are conventionally supplemented with DL-Methionine, which contains 50% D-Met and 50% L-Met. Nonetheless, only L-isomers are used in protein synthesis process, which forces the organism to convert all the isomers to L-form. In Met case, such conversion takes place in bird liver and kidney [2, 15, 16]. These arguments have been reinforcing the appeal for researches with the objective of evaluating the potential utilization of L-Met in replacement to DL-Met in poultry diets. The fact of whether Met sources influence or not the determination of the TSAA ratio in broiler/TSAA for broiler is unknown, which suggests that studies could focus on investigating such issue and answer this question. Additionally, besides the importance of determining the ideal Met:Cys/TSAA ratio for broilers, obtaining reliable information regarding the efficiency with which met is converted into Cys could help to achieve a clearer understanding of the dynamic of TSAA metabolism.

Studies involving labelled amino acid date from the last century [17] and since then, so many innovation have been observed in this area of science, given the improvements and developments of techniques, which allowed labelling the most varied molecules and track their final fates. Based on all these arguments, the current research was conducted in order to determine the optimum proportion between Met and CysTSAA in the total of sulfur amino acids (Met:Cys/TSAA ratio) in diets supplemented with different sources of Met for broilers in the grower feeding phase and to estimate the efficiency with Met is converted into Cys in broilers using labelled methionine and cystine.

## Results

### Performance assay

Irrespective of the performance response evaluated, no interactions were noticed between dietary Met:Cys/TSAA ratios and Met sources (*P* > 0.05). Methionine sources did not affect broiler performance, and thereby means are presented in Table 1 as the average of both sources. Broiler performance responses to Met:Cys/TSAA ratios and Met sources are detailed in Tables 1 and 2. Feed intake was unaffected by Met:Cys/TSAA ratios. Broilers fed 44:56 Met:Cys/TSAA ratio had the lowest BWG compared with the other dietary treatments (*P* < 0.05). Broilers fed 44:56 Met:Cys/TSAA ratio had the poorest FCR compared with broilersbroilers fed 50:50, 53:57, and 56:44 Met:Cys/TSAA ratios (P < 0.05), but no difference was noticed from broilers fed TSAA47:53 Met:Cys/TSAA ratio. Curvilinear-plateau regression model was fitted to data with the purpose of estimating the TSAA ratio in broiler/TSAA ratio in broiler diets.

**Table 1:** Body weight gain (BWG), feed intake (FI) and feed conversion ratio (FCR) from 14 to 28d of age after combination of sources

Met:Cys/TSAA ratio	BWG (g)	FI (g)	FCR (g/g)
44:56	912 ± 28.46 b	1469 ± 88.79	1.56 ± 0.10 b
47:53	970 ± 21.24 a	1482 ± 48.99	1.53 ± 0.05 ab
50:50	993 ± 23.71 a	1499 ± 62.88	1.51 ± 0.06 a
53:47	1001 ± 25.76 a	1482 ± 33.52	1.48 ± 0.06 a
56:44	1006 ± 24.24 a	1519 ± 63.01	1.51 ± 0.06 a
P-value			
Proportions	< 0.05	0.35	< 0.05
Sources	0.73	0.21	0.36
Proportions x Sources	0.96	0.84	0.97

Means in columns followed by distinct letters are different by the Tukey test (P < 0.05)

**Table 2:** Body weight gain (BWG), feed intake (FI) and feed conversion ratio (FCR) and their respective standard deviations for broilers fed different Met sources (L and DL) and Met:Cys/TSAA ratios

	14 to 28 days		
Met:Cys/TSAA			
L-Cys	BWG (g)	FI (g)	FCR (g/g)
44:56	912 ± 28.46	1466.50 ± 59.95	1.56 ± 0.07
L-Met/L-Cys			
47:53	970 ± 23.46	1489 ± 40.02	1.53 ± 0.03
50:50	991 ± 23.37	1487 ± 29.70	1.50 ± 0.04
53:47	1001 ± 30.37	1486 ± 39.90	1.49 ± 0.07
56:44	1013 ± 21.13	1546 ± 62.03	1.53 ± 0.07
DL-Met/L-Cys			
47:53	970 ± 21.03	1458 ± 44.00	1.50 ± 0.04
50:50	994 ± 26.26	1485 ± 60.61	1.49 ± 0.05
53:47	1001 ± 23.18	1478 ± 28.99	1.48 ± 0.05
56:44	1007 ± 24.20	1491 ± 55.89	1.49 ± 0.05
*P*-value			
Proportions	< 0.05	0.35	< 0.05
Sources	0.73	0.21	0.36
Proportions x Sources	0.96	0.84	0.97

### Labelled stable isotope assay

The data used to calculate the rates with which Met is converted into intermediates and Cys are detailed in Tables 3, 4 and 5. The recoveries of metabolic tracers (^15^N L-Met and ^15^N L-Cys) in the body, breast, liver, feather, and excreta samples are expressed in percentage and therefore, the effect of body weight variation among the animals was disregarded (Eqs. 2, 3 and 4). The recovery of stable nitrogen isotope recovery L-(^15^N) Met and L-(^15^N_2_) Cys were close to 100% in L-(^15^N)-Met supplemented-diets, whilst in L-(^15^N)-Cys these values were approximately 90% (Table 3). The analysis of aminogram in the different tissues and excreta of broilers fed L-(^15^N) Met supplemented-diets indicated very similar Met recovery values irrespective of the ratio assessed, being in average approximately 86% (Table 4). Conversely, Cys recoveries in broilers fed L-(^15^N) Cys supplemented-diets ranged from 93 to 111% as dietary Cys content decreased (Table 4). The values of methionine-specific ^15^N recovery in broilers given bird given L-(^15^N) Met supplemented-diets are detailed in Table 5. Broilers fed diets with the highest Cys content (44:56 and 50:50 Met:Cys/TSAA ratio) exhibited a narrow difference in Met recovery (66–68%), whilst this value in broilers fed 56:44 Met:Cys/TSAA ratio was 59%. When comparing Met and Cys deposition in the analyzed samples, both stable nitrogen isotope and aminogram analysis indicated that higher amounts of Met were incorporated into breast muscle protein compared with Cys, whilst the major deposition of Cys occurred in feathers. The rates with which Met was converted into intermediates and Cys is detailed in the Table 6. When Met:Cys/TSAA ratio increased from 44:56 to 56:44, the amount of Met converted into Cys increased from 27 to 43%, and the Met conversion into intermediates of Met cycle decreased from 24 to 12%.

**Table 3:** ^15^N recoveries from stable nitrogen isotope analysis

δ^15^N	^15^N intake (mg)	Body	Breast	Liver	Feather	Excreta	Total	Rec (%)
		^15^N retention (^15^N_R_, mg)						
L-(^15^N)-Met								
44:56	10.73 ± 0.06	6.21 ± 0.35	1.55 ± 0.05	0.37 ± 0.02	0.49 ± 0.02	2.12 ± 0.11	10.74 ± 0.48	100 ± 4
50:50	10.27 ± 0.28	5.83 ± 0.12	1.80 ± 0.13	0.36 ± 0.01	0.40 ± 0.05	1.85 ± 0.07	10.23 ± 0.20	100 ± 1
56:44	10.25 ± 0.49	5.53 ± 0.42	1.67 ± 0.10	0.29 ± 0.03	0.47 ± 0.04	1.85 ± 0.15	9.80 ± 0.49	96 ± 9
L-(^15^N_2_)-Cys								
44:56	12.06 ± 0.10	4.42 ± 0.26	1.04 ± 0.11	0.28 ± 0.04	2.18 ± 0.93	2.47 ± 0.23	10.39 ± 0.72	86 ± 6
50:50	11.58 ± 0.43	4.21 ± 0.70	1.07 ± 0.11	0.31 ± 0.05	2.71 ± 0.22	2.31 ± 0.14	10.60 ± 0.69	91 ± 4
56:44	11.79 ± 0.27	4.07 ± 0.29	1.07 ± 0.08	0.26 ± 0.01	2.66 ± 0.49	2.74 ± 0.50	10.79 ± 0.74	92 ± 8

**Table 4:** Amino acid recoveries from aminogram analysis

Aminogram	AA intake (mg)	Body	Breast	Liver	Feather	Excreta	Total	Rec (%)
		AA retention (AA_R_, mg)						
Met retention								
44:56	5063 ± 176	2287 ± 106	675 ± 20	89 ± 6	676 ± 60	605 ± 45	4332 ± 70	86 ± 4
50:50	4917 ± 288	2110 ± 145	786 ± 49	88 ± 3	575 ± 10	573 ± 30	4132 ± 166	84 ± 1
56:44	4701 ± 451	2084 ± 163	724 ± 71	66 ± 7	694 ± 30	543 ± 59	4114 ± 196	88 ± 5
Cys retention								
44:56	4590 ± 131	1781 ± 92	412 ± 12	61 ± 4	869 ± 128	1146 ± 85	4268 ± 58	93 ± 3
50:50	4081 ± 399	1652 ± 126	425 ± 30	64 ± 2	1040 ± 22	996 ± 57	4176 ± 139	103 ± 2
56:44	4211 ± 234	1790 ± 142	483 ± 42	52 ± 5	1120 ± 64	1212 ± 113	4659 ± 165	111 ± 8

**Table 5:** 15 N-Met recoveries from compound-specific stable isotope (CSIA) analysis

δ^15^N-Met^a^	^15^N-Met intake (mg)	Body	Breast	Liver	Feather	Excreta	Total	Rec (%)
		^15^N-Met retention (^15^N-Met_R,_ mg)						
L-(^15^N)-Met								
44:56	107.41 ± 0.58	38.54 ± 1.71	7.04 ± 0.07	1.78 ± 0.20	4.94 ± 0.40	21.19 ± 1.65	73.50 ± 1.42	68.4 ± 1
50:50	102.80 ± 2.77	35.16 ± 1.71	7.30 ± 0.13	1.70 ± 0.06	3.87 ± 0.20	20.01 ± 0.87	68.04 ± 1.73	66.2 ± 0
56:44	102.59 ± 4.90	31.31 ± 2.34	6.43 ± 0.27	0.75 ± 0.50	4.09 ± 0.51	18.44 ± 1.04	61.02 ± 3.19	59.6 ± 4

^a^ Results from compound-specific stable isotope analysis using labelled methionine (CSIA)

**Table 6:** Methionine conversion to cystine and intermediates

Met:Cys/TSAA ratio	^15^N_Rec_^a^	^15^N-Met_Rec_^b^	Met_Cys + interm._^c^	AA_Rec_ _(Cys)_^d^	^15^N_Rec__(Cys)_^e^	Cys_Met_^f^	Met_interm._^g^
	(%)						
44:56	100 ± 3.89	49 ± 4.62	51 ± 2.83	93 ± 10.92	66 ± 2.94	27 ± 7.83	24 ± 8.60
50:50	100 ± 2.80	47 ± 4.62	53 ± 2.41	103 ± 10.52	72 ± 3.89	31 ± 4.31	22 ± 3.61
56:44	96 ± 2.76	42 ± 3.77	54 ± 1.71	111 ± 9.50	68 ± 7.73	43 ± 4.11	12 ± 6.31

^a 15^N_Rec_ (Table 3) from Met

^b 15^N-Met_Rec_ (Table 5) excluding excreta

^c^ Met_Cys + interm_ = ^15^N_Rec (Met)_ - (^15^N-Met_Rec_)

^d^ AA_Rec_ (Table 4) of Cys

^e 15^N_Rec_ (Table 3) from Cys, excluding excreta

^f^ Cys_Met_ = AA_Rec (Cys)_ - ^15^N_Rec (Cys)_

^g^ Met_interm._ = Met_Cys + interm._ - Cys_Met_

## Discussion

The current research was conducted with the purpose of establishing the optimum proportion between Met and Cys (Met:Cys/TSAA ratio) for broilers from to 14 to 28 d of age. Since the metabolism of Cys is particularly dependent on Met supply, it was expected that some biological events would occur in TSAA metabolism in face of the changes in the dietary supply of Met and TSAACys. Therefore, inTSAA parallel, a labelled isotope study was conducted to provide information to support the performance outcomes obtained and to understand the metabolic pathways in the organism in which Met is involved after consumed. In dose-response assays, the success in obtaining reliable estimates of nutrient requirements, among several factors, depends on the range of the levels of the nutrient studied, i.e. the optimal dose of a specific nutrient can only be estimated if the levels studied allow observing different phases of growth response. Additionally, as pointed out by Garlich et al. [18], there is the inherent decrease in sensitivity of the assay as the diets approach nutritional adequacy. Based on the latter assumption, in the current research, the amount of TSAA provided by experimental diets was fixed in 70% of the TSAA recommended by [2] and such diets differed only with respect of the Met:Cys/TSAA ratio assessed and the Met source supplemented.

In general, methionine is supplemented in poultry diets as DL-methionine, which consists of the mixture of 50% D-Met and 50% L-Met. It is well acknowledged that the organism incorporates only L-isomers into bodily proteins, which makes the conversion of the D-isomer into the L-isomer by the organism a mandatory step for the synthesis of protein [19]. Such conversion of isomers takes place in the liver and kidneys in metabolic pathways, whose effectiveness do not achieve the rate of 100% [20–25]. Considering such losses of efficiency, several research efforts have been put forward finding and validating alternatives sources to DL-Met with a higher bioavailability. Based on recent evidences published in literature that L-Met has a higher relative bioavailability than DL-Met, and that broilers fed L-Met supplemented-diets had greater performance than those fed DL-Met, this study was conducted based on the hypothesis that L-Met could indeed improve performance responses [26, 27]. However, contrary to what was previously hypothesized the Met sources tested herein did not affect broiler overall performance (*P* > 0.05). These outcomes support previous research that broilers and turkeys at different ages, fed either L or DL-Met supplemented-diets, did not differ from each with respect to performance responses [18, 28, 29].

### Optimum Met:Cys/TSAA ratio

The outcomes obtained from the current research TSAA clearly indicate that the range among the ratios were indeed sufficient to induce a response, which in turn allowed fitting statistical models to collected performance data which allowed estimating ideal Met:Cys/TSAA ratio for broilers. Graber and Baker [30] highlight that the complexity of determining nutrient requirements relies on the difference in the estimates produced from statistical models. With a considerable range in the dose of a nutrient, broiler responses exhibit different phases: an increase in response, a plateau phase, and a decrease in performance, which in some cases are associated with incidence of mortality if the dose administrated exceeds the toxicity tolerance of the organism [31]. The model should therefore describe as close as possible the entire pathway at which an animal move towards its maximum response to the range of a given nutrient. Initially, collected performance data obtained in this research were fitted to polynomial quadratic, linear-plateau and curvilinear-plateau regression models. After fitting data, AIC value indicated that data fitted better to polynomial quadratic model. However, such model was ignored since when estimating requirements it assumes a bilateral symmetry in animal responses as a function of increments in the nutrient intake, which could not be biologically condoned. Estimates from curvilinear-plateau regression modelTSAA were accepted as those which better represented the responses obtained in this research. Curvilinear-plateau estimated the breakpoint for both BWG and FCR at 52:48 Met:Cys/TSAA ratio. These outcomes are consistent and support the 55:45 Met:Cys/TSAA ratio for broilers in the starter phase recommended by the [2]. Research efforts with the purpose of investigating the effects of TSAA in broiler performance usually range the levels of Met + Cys through supplementing a crystalline Met source. When both assays described herein were designed and conducted until the present date, no investigations in which the total amount of TSAA was fixed so that Met and Cys could be supplied in order to range the proportion between both amino acids were conducted. Such lack of references makes the current research innovative at some point, but concomitantly it makes harder any kind of comparisons with other results.

It was hypothesized that when supplemented in diets, Cys could spare dietary Met, which in turn could be used for body and feather protein accretion instead of being converted into Cys. However, contrary to such expected positive effects, as L-Cys was added to diets, i.e. as Met:Cys/TSAA ratio decreased, broiler performance was impaired, which indicates that broilers need to convert Met into Cys, irrespective whether diets are supplemented or not with L-Cys. Cysteine may be originated from diet, muscle proteolysis and/or in the liver from dietary Met [32]. Due to its unstable nature, it may be readily autoxidizes to cystine, which in turn is the most abundant amino acid in feather keratin. For modern broiler industry, poor feathering has been correlated to low carcass quality and carcass condemnations at slaughtering [33, 34]. Considering that broilers are generally slaughtered around the 42 day of age, the period in which feather growth rate reaches maximum values comprises 14 to 28 d post hatch. Until 28 d of age, it is expected a complete replacement of the primary feathers by the secondary ones. Since protein accretion rates in feathers are increased, higher requirements for amino acids, particularly Cys, is also expected in this period [35]. Why L-Cys supplementation or Met deficiency decreased broiler performance instead of improving it, it is still unknown. However, two hypothesis, which explain such phenomenon may be risen. The first one considers the premise that the presence of Cys in plasma may presumably act as a trigger, which could signalize to the organism that Met does not need to absorbed and then converted into Cys, since Cys status is in accordance to organism needs, i.e. Cys may downregulate the intestinal uptake of Met. Although, this hypothesis makes some sense, data collected herein cannot support or corroborate its validation. Another hypothesis to explain the performance impairments implies in considering the dynamic and importance of the Met cycle for metabolism. Although simple, the Met cycle is crucial to maintain structural and metabolic functions of the organism. The first step of this cycle consists of the formation of the derivate S-adenosilmethionine (SAM), a methyl donor, which enables the occurrence of a plethora of transmethylation reactions, which include the DNA methylation, and the synthesis of phosphatidylcholine, creatine and polyamines. In a next step, homocysteine is formed from SAM and from this step on it may follow distinct pathways: 1) being remethylated to methionine through the transfer of a methyl group from betaine by the enzyme betaine-homocysteine methyl transferase to produce dimethylglycine or via 5-methyl tetrahydrofolate [36] in a process dependent on vitamin B12; and/or 2) being catabolized to form cysteine in a vitamin-B6-dependent pathway of transsulfuration where sulfur group of homocysteine is transferred to serine [37, 38]. Since the first step of Cys synthesis involves the occurrence of methylation reactions in the Met cycle, and that these reactions are a salvage mechanism to ensure the maintenance of vital processes, if provided in low concentrations, insufficient amounts of Met would be available for optimum protein accretion rates, which could justify our results [39–42]. In other terms, a minimum amount of Met must participate on Met cycle so that the aforementioned reactions may occur in rates which warrant the adequate occurrence of secondary physiological process than growth.

Labelling amino acids with stable isotopes has proved to be a useful tool understand amino acid metabolism and, therefore, explain the results obtained in the performance assay. In so doing, the first focus of this research was to recover the amount of ^15^N supplied in diets in the body and excreta. These outcomes indicated that the recovery rates for ^15^NMet from Met in the in broilers fed 44:56, 50:50, and 56:44 Met:Cys/TSAA ratios was equal to 100, 100, and 96%, respectively. These higher recovery rates corroborate the effectiveness of 15 N as tracer in studies involving labelled molecules. Thereafter, it was determined specifically the labelled Met (^15^N-Met) incorporated into body, feather, and liver protein, which after subtracted by the recovery rates above cited resulted in conversion rate of Met into Cys/intermediates (MetCys+interm) of 51, 53, and 54% for the diets containing 44:56, 50:50, and 56:44 Met:Cys/TSAA ratios, respectively. Whilst data collected in the current study could not support the first hypothesis raised above, the outcomes from the labelled isotopes assay provided some evidences, which prove to be true the second hypothesis raised about why L-Cys supplementation impaired broiler performance,. The fact that the conversion rates of Met into Cys/intermediate (Table 4) were very similar among dietary treatments clearly demonstrates that Met was equally converted into Cys and intermediates of Met cycle, regardless of the amount of dietary L-Cys supplied. From this answer, however, another question raises: were the Met:Cys/TSAA ratios studied herein capable of influencing the proportion of Met converted into intermediates of Met cycle and Cys?TSAAbroilersTSAA.

A detailed in Table 6, the lowest the Cys content of diets, the highest was the amount Met converted into Cys and the lowest was Met conversion into intermediates of Met cycle. The amount of Cys originated from Met (Cys_Met_) decreased from 43 to 27% when dietary Met:Cys/TSAA ratio ranged from 56:44 to 44:56, i.e. when L-Cys supplementation increased. Once Cys was already supplied by diets, dietary Met used in transmethylation pathways increased, which can be confirmed by the increase in the amount of intermediates originated from Met (Met_interm._) when Met:Cys/TSAA ratios increased form 44:56 to 56:44. These outcomes prove that broilers utilized L-Cys from diets in which Cys content was lower. Additionally, it seems that Met will inevitably be used in Met cycle so that methyl transfer reactions ensure the adequate occurrence of the important physiological processes. It explains why broilers fed diets in which Met content was lower had lower performance responses compared with broilers fed higher Met supply: lower was the amount of Met available for body protein accretion.

## Conclusion

When Cys assumes a high proportion of total TSAA in broiler diets, dietary Met is not spared to be used in body protein accretion, but rather to be used in transmethylation pathways. Dietary Met:Cys/TSAA ratios for broiler from 14 to 28 d of age must be higher than 52:48.

## Methods

### Broiler performance assay

#### Broilers, experimental design, and housing

One-d-old male Cobb 500 broilers were obtained from a local commercial hatchery (Pluma Agroavicola, BR) and housed in an environmentally controlled room with a cellulose-pad cooling system associated with exhausters. From one to 14 d post-hatch broilers were fed a standard diet formulated to meet or exceed [2] recommendations. On d 14 post-hatch, a total of 1080 broilers were weighed and initial body was similar among treatment groups (average initial body weight of 0.390 ± 0.004 kg). Six replicate pens of 20 broilers were randomly assigned to each of nine treatments. A factorial arrangement (4 × 2) consisting of four Met:Cys/TSAA ratios (56:44, 53:47, 50:50, and 47:53) supplemented with two different Met sources (DL-Met or L-Met). An additional diet TSAAin which no sources of synthetic Met was added was formulated to contain a 44:56 Met:Cys/TSAA ratio. Each pen (1.4 m × 3.0 m) was equipped with six nipple drinkers and a tubular feeder, which provided water and experimental diets (mash form) on ad libitum basis. Photoperiod was daily set at 18 L:6D (light:dark) throughout the entire experimental period. Throughout the 14-d feeding assay, broilers were raised under thermoneutral conditions and environmental temperature and humidity were controlled as recommended by the strain guideline [43]. Environmental temperature to which broilers were exposed prior to the beginning of the trial were 31 °C ± 2 °C at the first week, 27 °C ± 1.5 °C at the 2nd week, and 24 °C ± 1.5 °C during the trial. Humidity were kept constant (65% ± 4%), from the 1st d of age until the end of the trial. The broilers used in the current research were used in a subsequent performance assay, which as well as this research had the protocols previously approved by the institutional Animal Care and Use Committee of the Faculdade de Ciências Agrárias e Veterinárias (UNESP, Jaboticabal, Brazil).

#### Experimental diets

Experimental diets were formulated to meet or exceed broiler nutrient recommendations [10] (Table 7), except for dietary total sulfur amino acid content, which was supplied at 0.65%, regardless of the experimental treatment, which corresponded to 70% of the recommendation of Rostagno et al. [2]. Such deficiency was provided in order to ensure that both amino acids would be limiting in diets. The changes in the amount of Met relative to Cys was obtained by changing the supplemented level of L-Cystine (100%) in relation to DL/L-Met (99%) and vice versa. Prior to diet formulation, all ingredients were analyzed for total amino acids content. Standardized ileal digestible amino acids were estimated based on coefficient of digestibility described by Rostagno et al. [2]. Crystalline amino acid were assumed to be 100% standardized ileal digestible (SID). – Once, TSAA metabolism is dependent on a series of methyl donors and acceptors, all diets had equal premix, and the same batches of ingredients were used.

**Table 7:** Ingredients and nutrient composition of experimental diets as-fed basis

Met:Cys/TSAA TSAAratio	Standart diet (1-14d)	44:56	47:53		50:50		53:47		56:44	
Ingredients (g.kg^−1^)		No Met	L-Met	DL-Met	L-Met	DL-Met	L-Met	DL-Met	L-Met	DL-Met
Corn (7.86%)	620.64	545.60	545.04	545.59	544.47	545.58	543.91	545.56	543.34	545.55
Soybean meal (45%)	216.68	350.03	350.10	350.03	350.17	350.03	350.24	350.03	350.31	350.04
Corn gluten meal (60%)	105.11									
Peanult meal (48.2%)		20.93	21.24	20.99	21.55	21.05	21.86	21.10	22.18	21.16
Soybean oil	9.27	45.81	46.00	45.77	46.19	45.73	46.37	45.68	46.56	45.64
Dicalcium phosphate	19.39	15.27	15.26	15.27	15.26	15.27	15.26	15.27	15.26	15.26
Limestone	9.31	9.04	9.04	9.04	9.04	9.04	9.04	9.04	9.04	9.04
Sodium bicarbonate	2.00	4.14	4.14	4.14	4.13	4.14	4.13	4.14	4.13	4.14
Salt	3.61	2.08	2.08	2.08	2.08	2.08	2.08	2.08	2.08	2.08
DL-Methionine (98%)	2.88	0.00	0.00	0.19	0.00	0.37	0.00	0.55	0.00	0.74
L-Methionine (99%)		0.00	0.18	0.00	0.37	0.00	0.55	0.00	0.73	0.00
L-Cystine (100%)		0.73	0.55	0.55	0.37	0.37	0.18	0.18	0.00	0.00
L-Lysine HCl (78%)	7.03	2.37	2.37	2.37	2.37	2.37	2.37	2.37	2.37	2.37
L-Threonine (98%)	1.58	0.93	0.93	0.93	0.93	0.93	0.93	0.93	0.93	0.93
L-Valine (96.5%)		0.47	0.47	0.47	0.47	0.47	0.48	0.47	0.48	0.47
Premix^a^	1.5	1.60	1.60	1.60	1.60	1.60	1.60	1.60	1.60	1.60
Choline Chloride (60%)	1.00	1.00	1.00	1.00	1.00	1.00	1.00	1.00	1.00	1.00
Nutrients content										
Met. energy (Mcal/Kg)	3.100	3.10 (2.9)	3.10 (2.9)	3.10 (2.9)	3.10 (2.9)	3.10 (2.9)	3.10 (2.9)	3.10 (2.9)	3.10 (2.9)	3.10 (2.9)
Crude protein (%)	22.0	21.5 (22)	21.5 (22)	21.5 (22)	21.5 (22)	21.5 (22)	21.5 (22)	21.5 (22)	21.5 (22)	21.5 (22)
Dig. Met+Cys (%)	0.953	0.65 (0.60)	0.65 (0.60)	0.65 (0.62)	0.65 (0.60)	0.65 (0.60)	0.65 (0.61)	0.65 (0.62)	0.65 (0.61)	0.65 (0.60)
Dig. Met (%)	0.639	0.29 (0.26)	0.31 (0.28)	0.31 (0.29)	0.33 (0.30)	0.33 (0.30)	0.34 (0.32)	0.34 (0.33)	0.36 (0.34)	0.36 (0.34)
Dig. Cys (%)	0.314	0.36 (0.34)	0.34 (0.32)	0.34 (0.33)	0.33 (0.30)	0.33 (0.30)	0.31 (0.29)	0.31 (0.29)	0.29 (0.27)	0.29 (0.26)
Dig. Lys (%)	1.324	1.19 (1.16)	1.19 (1.16)	1.19 (1.16)	1.19 (1.16)	1.19 (1.16)	1.19 (1.16)	1.19 (1.16)	1.19 (1.16)	1.19 (1.16)
Dig. Thr (%)	0.861	0.79 (0.75)	0.79 (0.75)	0.79 (0.75)	0.79 (0.75)	0.79 (0.75)	0.79 (0.75)	0.79 (0.75)	0.79 (0.75)	0.79 (0.75)
Dig. Val (%)	1.019	0.92 (0.89)	0.92 (0.89)	0.92 (0.89)	0.92 (0.89)	0.92 (0.89)	0.92 (0.89)	0.92 (0.89)	0.92 (0.89)	0.92 (0.89)

^a^ Content (per kg of diet) – vit. A = 10,575 UI; vit. D3 = 2554 UI; vit. K = 1.8 mg; vit. E = 14.87 mg; vit. B1 = 2.00 mg; vit. B2 = 4.5 mg; vit. B6 = 2.50 mg; vit. B12 = 2.00 mg; niacin = 30.00 mg; folic acid = 0.75 mg; calcium pantothenate = 11.74 mg; biotin = 0.01; iron = 43.44 mg; zinc = 43.35 mg; copper = 8.56 mg; manganese = 56.00 mg; iodine = 0,56 mg; selenium = 0,34 mg; antioxidant 4.20 mg; Salinomycin sodium 12%; Butil hidroxy toluene BHT. Values in parentheses indicate analyzed dietary concentration of the amino acids

#### Data collection

At 28 d of age all broilers and feeders were weighed to determine average daily feed intake (FI, g/bird) and body weight (BW, kg). Body weight gain (BWG, g/bird) and feed conversion ratio (FCR) were calculated from such data. Mortality was daily recorded to adjust feed intake and feed conversion ratio. Met and Cys intakes (mg/bird/day) were calculated from FI and the analyzed content of Met and Cys in the diet.

#### Statistical analysis

All collected performance data were analyzed as two-way ANOVA using the GLM procedure of SAS 9.0 (SAS Institute Inc., Cary, NC). The statistical model included the fixed main effects of dietary Met source (DL-Met or L-Met) and dietary Met:Cys ratio (65:35, 57:43, 50:50 and 43:57), as well as the interaction between both factors. Because interaction effects between Met source and Met:Cys ratio were not found for any of the evaluated parameters, data from the main effects were pooled and reanalyzed. Quadratic broken-line regression was computed by the PROC NLIN procedure of SAS [44] to estimate optimal dietary Met:Cys ratio based on performance traits and statistical differences were considered when *P* < 0.05.

### Labelled stable isotope assay

#### Husbandry, experimental design and experimental diets

On d 14 posthatch, twenty-one male broilers were weighed (0.390 ± 0.004 kg) and transferred to individual metabolic cages. Initially, the broilers were divided into two groups of nine broilers each (group I and II) and one group of three broilers. Group I and II received the feed with labelled amino acids L-(^15^N) Methionine and L-(^15^N_2_) Cystine, respectively, whilst group III consisted of a reference group of 3 broilers euthanized with carbon dioxide (compressed cylinder, 100% CO_2_) asphyxiation on day zero (14 d posthatch), and all efforts were made to minimize suffering. The gas was brought into the cage with a flow rate of 250 L/min for 15 min. Birds were not anaesthetized prior to euthanasia. Regarding group I, three individual replicate cages were randomly assigned to three different Met:Cys (44:56, 50:50, and 56:44) in diets daily enriched with a fixed-dose of labelled L-Met (60 mmol.kg^− 1^). The same procedure was replicated with broilers of group II, however the labelled amino acid was L-Cys (35 nmol.kg^− 1^). All diets above described consisted of the same treatments used in the dose-response assay. Throughout the 14-d feeding assay broilers were housed into stainless steel metabolic cages (0.5 × 0.5 m) in a controlled environmental room and had ad libitum access to water and feed (mash form). Throughout the assay broilers were exposed to the environmental temperature of 25 °C ± 2 °C and the humidity of 63% ± 2%. Lighting program was set at 18 L:6D (light:dark).

#### Enrichment with labelled amino acids

Both groups I and II were fed diets containing the Met:Cys ratios of 44:56, 50:50, 56:44, which were the same tested in the performance trial. The difference between the referred groups was the labelled amino acid added to diets. Therefore, broilers from group I, regardless of the Met:Cys tested, received diets enriched with L-(^15^N) Methionine, whilst diets in group II was enriched L-(^15^N_2_) Cystine and the enrichment procedure was performed daily by feed with a fixed-dose according to the weight of the bird (60 and 35 mmol.kg^− 1^, respectively). The L-(^15^N) Met (150.21 g.mol^− 1^, 96–98 atm% abundance, Cambridge Isotope Laboratories®) was diluted in deionized water (100 mL) in a graduated reagent bottle and then, stored under refrigeration at 5 °C. The L-(^15^N_2_) Cys (242.29 g.mol^− 1^, 98 atm% abundance, Cambridge Isotope Laboratories®), due to its insolubility in water, was weighed on a precision analytical balance and supplemented in the crystalline form. The dose (in μL.kg^− 1^) for each bird was calculated according to the the following Eq. 1:

1 $$ {\mathbf{Qtd}}_{\mathbf{aa}}=\left({{\mathbf{D}}_{\mathbf{aa}}}^{\ast}{\mathbf{BW}}^{\ast}{\mathbf{MW}}^{\ast}\mathbf{Wast}\right)/\left(\mathbf{10}{\mathbf{00}}^{\ast}\mathbf{C}\right) $$

where D_aa_ is the labelled amino acid dose relative to the addition of ^15^N amino acid (μmol); BW is the weight of the animal (kg); MW is the molecular weight of the labelled amino acid; Wast is an increase of 3% assuming a waste of feed by broilers; the value 1000 is used as the conversion factor from micrograms to milligrams; C is the concentration of labelled amino acid (mg.mL^− 1^). It is possible to transform mL into μL after this calculation by multiplying the Qtd_aa_ value by 1000 and, thus determining the dose (μL.kg^− 1^). Initially, the broilers were weighed to obtain the BW value and calculate the amount of labelled amino acid based on Eq. 1. This amount was supplied daily in the feed at various points using micropipettes of variable volume (10 to 100 μL; 100 to 1000 μL and 1000 μL to 10,000 μL) and mixed with a single plastic spoon per bird for a better homogenization. The amounts of labelled Met and labelled Cys were supplied based on [16, 20], respectively. Labelled amino acids were not considered as dietary Met or Cys, both amino acids were provided in a very low concentration.

#### Measurements

At 14d of age, three broilers were euthanized with carbon dioxide(compressed cylinder, 100% CO_2_) asphyxiation, and all efforts were made to minimize suffering. The gas was brought into the cage with a flow rate of 250 L/min for 15 min. Birds were not anaesthetized prior to euthanasia. Enrichment with the labelled amino acid was performed daily until the end of the experimental period (28d of age). At 28d of age, broilers were euthanized with carbon dioxide (compressed cylinder, 100% CO_2_) asphyxiation, and all efforts were made to minimize suffering. The gas was brought into the cage with a flow rate of 250 L/min for 15 min. Birds were not anaesthetized prior to euthanasia. The birds were used to determine ^15^N deposition in body, breast muscle, liver and feathers. As well as at 14d of age, birds were not anaesthetized prior to euthanasia. Excreta were daily collected and at the end of the assay, a pool for each cage was homogenized to estimate ^15^N losses. Body samples corresponded to the remaining tissues than those above mentioned (digesta content was manually removed). All tissues were weighed and analyzed separately.

#### Sample preparation and chemical analysis

The samples of body, breast muscle, liver, feathers and excreta were dried in a freeze-drier (Edwards 501, Thermo) for 72 h, under 800 mbar pressure. All samples were frozen by nitrogen liquid and grounded in cryogenic mill (SPEX SamplePrep 2010 Geno/Grinder 2010) at -196 °C during 3 min to obtain homogeneity [17]. The samples, weighed and packed in tin capsules, were burned in a combustion furnace of the elemental analyzer coupled to the mass spectrometer (Thermo Fisher Scientific, Waltham, MA, USA) to determine the isotope concentration and percentage of nitrogen. The isotope concentrations were expressed in atm%^15^N, with permissible analytical error of 0.1‰. The determination of labelled Met was performed by the derivatization and normalization procedures in the compound-specific stable isotope analysis of nitrogen in amino acids as described by [45].

#### Isotope/amino acid recoveries and methionine conversion

The ^15^N retention (^15^N_R_) was calculated based on ^15^N intake ^15^N content of breast tissue, liver, feathers, excreta, and the remaining part of carcass as follows:

2 $$ {}^{15}{\mathrm{N}}_{\mathrm{R}}={\mathrm{DM}}^{\ast}\%{\mathrm{N}}^{\ast}\left[\left({\mathrm{Ab}}_{\mathrm{p}}-{\mathrm{Ab}}_{\mathrm{nat}}\right)/\left({\mathrm{Ab}}_{\mathrm{aa}}-{\mathrm{Ab}}_{\mathrm{nat}}\right)\right] $$

Where ^15^N_R_ was the ^15^N retention (mg) from labelled amino acid; DM was the dry mass of the sample (mg); %N was the nitrogen content (%); Ab_p_ was the isotope abundance of enriched tissue; Ab_nat_ was the isotope abundance of non-enriched tissue (determined at 14d of age); Ab_aa_ was the isotope abundance of the L-(^15^N) amino acid.

The same equations were applied for the CSIA results, only replacing δ^15^N by methionine-specific δ^15^N, as presents in Eq. 3:

3 $$ {}^{15}\mathrm{N}-{\mathrm{Met}}_{\mathrm{R}}={\mathrm{DM}}^{\ast}\%{\mathrm{Met}}^{\ast}\left[\left({\mathrm{Ab}}_{\mathrm{pCSIA}}-{\mathrm{Ab}}_{\mathrm{natCSIA}}\right)/\left({\mathrm{Ab}}_{\mathrm{aa}}-{\mathrm{Ab}}_{\mathrm{natCSIA}}\right)\right] $$

Where ^15^N-Met_R_ was the ^15^N-Met retention (mg) from L-(^15^N) Met; DM was the dry mass of the sample (mg); %Met was the Met content (%); Ab_pCSIA_ was the ^15^N-Met abundance of enriched tissue; Ab_natCSIA_ was the ^15^N-Met abundance of non-enriched tissue (determined at 14d); Ab_aa_ was the isotope abundance of the L-(^15^N) Met.

The amino acid retention was calculated using the aminogram results (CBO lab, Brazil), according to the Eq. 4:

4 $$ {\mathrm{AA}}_{\mathrm{R}}=\left({\mathrm{DM}}^{\ast}\%\mathrm{AA}\right)-{\mathrm{AA}}_{\mathrm{R}}\mathrm{i} $$

Where AA_R_ was the amino acid retention; DM was the dry mass of the sample (mg); %AA was the amino acid content (%) and AA_R_i was the amino acid retention before the experimental period.

Once calculated, the retention rates were used to calculate recovery rates of Met and Cys. The isotope/amino acid recoveries values were determined by dividing the total isotope/amino acid retention by the total isotope/amino acid intake.

5 $$ \mathrm{Rec}=\left({\mathrm{X}}_{\mathrm{R}}/{\mathrm{X}}_{\mathrm{I}}\right)\ast 100 $$

Where Rec is the recovery rate; X_R_ is the retention of Met or Cys calculated based on the Eqs. 2, 3, or 4 and X_I_ is the intake of ^15^N, methionine-specific ^15^N, or Met and Cys.

The conversion of Met into intermediates and Cys (Met_Cys + interm_) was calculated based on the difference between the recovery rates of ^15^N (^15^N_Rec_ (Met)) and methionine-specific ^15^N (^15^N-Met_Rec_) as follows:

6 $$ {\mathrm{Met}}_{\mathrm{Cys}+\mathrm{interm}}={}^{15}{\mathrm{N}}_{\mathrm{Rec}}\ \left(\mathrm{Met}\right)-\left({}^{15}\mathrm{N}-{\mathrm{Met}}_{\mathrm{Rec}}\right) $$

The amount of Met converted into Cys (Cys_Met_) were calculated based on the difference between the recovery of Cys using the aminogram analysis (AA_Rec_ (Cys)) and the recovery of ^15^N from Cys (^15^N_Rec_ (Cys)) as follows:

7 $$ {\mathrm{Cys}}_{\mathrm{Met}}={\mathrm{AA}}_{\mathrm{Rec}}\ \left(\mathrm{Cys}\right) -^{15}{\mathrm{N}}_{\mathrm{Rec}}\ \left(\mathrm{Cys}\right) $$

The amount of Met converted into intermediates of Met cycle (Met_interm._) was calculated based on the difference between the conversion of Met into intermediates and Cys (Met_Cys + interm_) detailed in Eq. 6 and the amount Met converted into Cys (Cys_Met_) as detailed in Eq. 7:

8 $$ {\mathrm{Met}}_{\mathrm{interm}.}={\mathrm{Met}}_{\mathrm{Cys}+\mathrm{interm}.}-{\mathrm{Cys}}_{\mathrm{Met}} $$

## Abbreviations

BWG: Body weight gain
Cys: Cystine
FCR: Feed conversion ratio
FI: Feed intake
Met: Methionine
TSAA: Total sulfur amino acids
